# Adoption strategies of fertility-sparing surgery for early-stage cervical cancer patients based on clinicopathological characteristics: a large retrospective cohort study

**DOI:** 10.3389/fsurg.2024.1456376

**Published:** 2024-08-22

**Authors:** Ying Ning, Xinyan Gao, Yan Kong, Yan Wang, Tian Tian, Yu Chen, Yufei Yang, Ke Lei, Zhumei Cui

**Affiliations:** ^1^ Department of Clinical Medicine, Qingdao University, Qingdao, China; ^2^ Department of Obstetrics and Gynecology, The Affiliated Hospital of Qingdao University, Qingdao, China; ^3^ Center of Tumor Immunology and Cytotherapy, Medical Research Center, The Affiliated Hospital of Qingdao University, Qingdao, China

**Keywords:** early-stage cervical cancer, fertility preservation, local excision, hysterectomy, prognosis

## Abstract

**Background:**

The demand for fertility-sparing surgery (FSS) is increasing among patients with early-stage cervical cancer (CC). This study aimed to evaluate the feasibility of local excision as an alternative to hysterectomy in stage I CC patients aged 15–39 years—commonly referred to as adolescents and young adults (AYAs)—with varying clinicopathological characteristics.

**Methods:**

Using the Surveillance, Epidemiology, and End Results (SEER) database, we identified patients diagnosed between 2000 and 2020. We examined treatment interventions across different age groups, degrees of histological types, tumor differentiation, and tumor stages. The effect of local excision vs. hysterectomy was assessed by comparing overall survival (OS) and disease-specific survival (DSS) rates.

**Results:**

A total of 10,629 stage I AYA cervical cancer patients were included in this study. Among these patients, 24.5% underwent local excision for fertility preservation, while 67.3% underwent radical hysterectomy. For patients with cervical squamous cell carcinoma (SCC), long-term outcomes favored local excision over hysterectomy, and a similar trend was observed in those with adenosquamous cell carcinoma (ASCC). However, the prognosis was comparable among patients with cervical adenocarcinoma (AC). In patients with well- and moderate- differentiated tumors, local excision demonstrated superior OS compared to hysterectomy. No significant differences in prognosis were found between the two surgical interventions for patients with poorly differentiated and undifferentiated tumors. In stage IA patients, local excision was considered a viable alternative to hysterectomy. In stage IB1–IB2, FSS yielded prognostic outcomes comparable to those of hysterectomy. Conversely, patients with stage IB3 exhibited significantly shorter 5-year OS and DSS following local excision than those who underwent hysterectomy.

**Conclusion:**

In stage IA–IB2 (diameter ≤4 cm) AYA patients, local excision may serve as a viable option for fertility preservation. The histological type of SCC, AC, and ASCC, along with differentiation, should not serve as restrictive factors in determining fertility preservation strategies for these patients. Patients with early-stage, well- or moderately-differentiated SCC may benefit from local excision surgery, even when fertility preservation is not the primary objective.

## 1 Introduction

Cervical cancer (CC) is the most prevalent malignancy of the female reproductive system, with significant global health implications (1). In 2022, there were an estimated 662,301 new cases and 348,874 deaths attributed to cervical cancer worldwide (2). Among these figures, individuals aged 15–39 years—commonly referred to as adolescents and young adults (AYAs)—accounted for 105,728 new cases and 32,575 deaths, making CC the third most common cancer and the second leading cause of cancer-related mortality among young females (2). Despite the implementation and increasing uptake of cancer initiatives in numerous countries, the incidence of CC among young women has shown a troubling upward trend in certain regions in recent years (3).

For patients diagnosed with early-stage CC, the standard treatment is radical hysterectomy with or without pelvic lymph node dissection. The 5-year survival rate for stage I CC patients exceeds 90% (4). Currently, the focus of treatment for young patients has shifted from solely improving survival rates to enhancing quality of life (5). Given that the AYA demographic encompasses prime reproductive years, fertility preservation is a crucial consideration for maintaining a satisfactory quality of life. As societal trends increasingly lead young women to marry later in life, many of these individuals express a strong desire to conceive following a cancer diagnosis (6–9). Moreover, studies have indicated that the loss of fertility in women with a history of gynecological malignancies can adversely affect their mental health and sexual function (10). Consequently, preserving fertility in AYA cervical cancer patients has emerged as a significant challenge.

For those patients who prioritize fertility preservation, available surgical options for fertility-sparing surgery (FSS) include conization or simple cervical excision, as well as (vaginal or abdominal) radical trachelectomy. However, the feasibility of FSS is influenced by various factors, such as age at diagnosis, tumor pathological characteristics, and International Federation of Gynecology and Obstetrics (FIGO) stage. While extensive follow-up studies at several medical institutions have substantiated the efficacy of local excision to some extent, comprehensive research offering a broad spectrum of FSS options across diverse patient populations is lacking. Thus, there is an urgent need for extensive studies to evaluate the safety of local excision compared with hysterectomy in patients with varying clinicopathological characteristics (11). This study endeavored to investigate the treatment modalities for stage I AYA cervical cancer patients, and to assess the safety of local excision as an alternative to hysterectomy across different patient profiles. By doing so, we seek to establish a framework for the personalized selection of FSS, thereby providing a theoretical foundation for clinical decision-making in this patient population.

## 2 Materials and methods

### 2.1 Data source

The data for this study were sourced from the Surveillance, Epidemiology, and End Results (SEER) database, a resource supported by the National Cancer Institute (NCI) of the United States. Encompassing approximately 30% of the U.S. population, the SEER database furnishes comprehensive data on patient demographics, tumor-related characteristics, diagnosis, treatment modalities, and subsequent follow-up. For the purposes of this study, the SEER*Stat 8.4.3 version was utilized to aggregate data from patients spanning the period from 2000 to 2020. The SEER database upholds stringent measures to safeguard patient confidentiality, and research conducted utilizing this database is not contingent upon obtaining ethical approval post-application review. Importantly, this study adhered to the principles outlined in the Helsinki Declaration.

### 2.2 Study cohort selection

**Figure 1:**
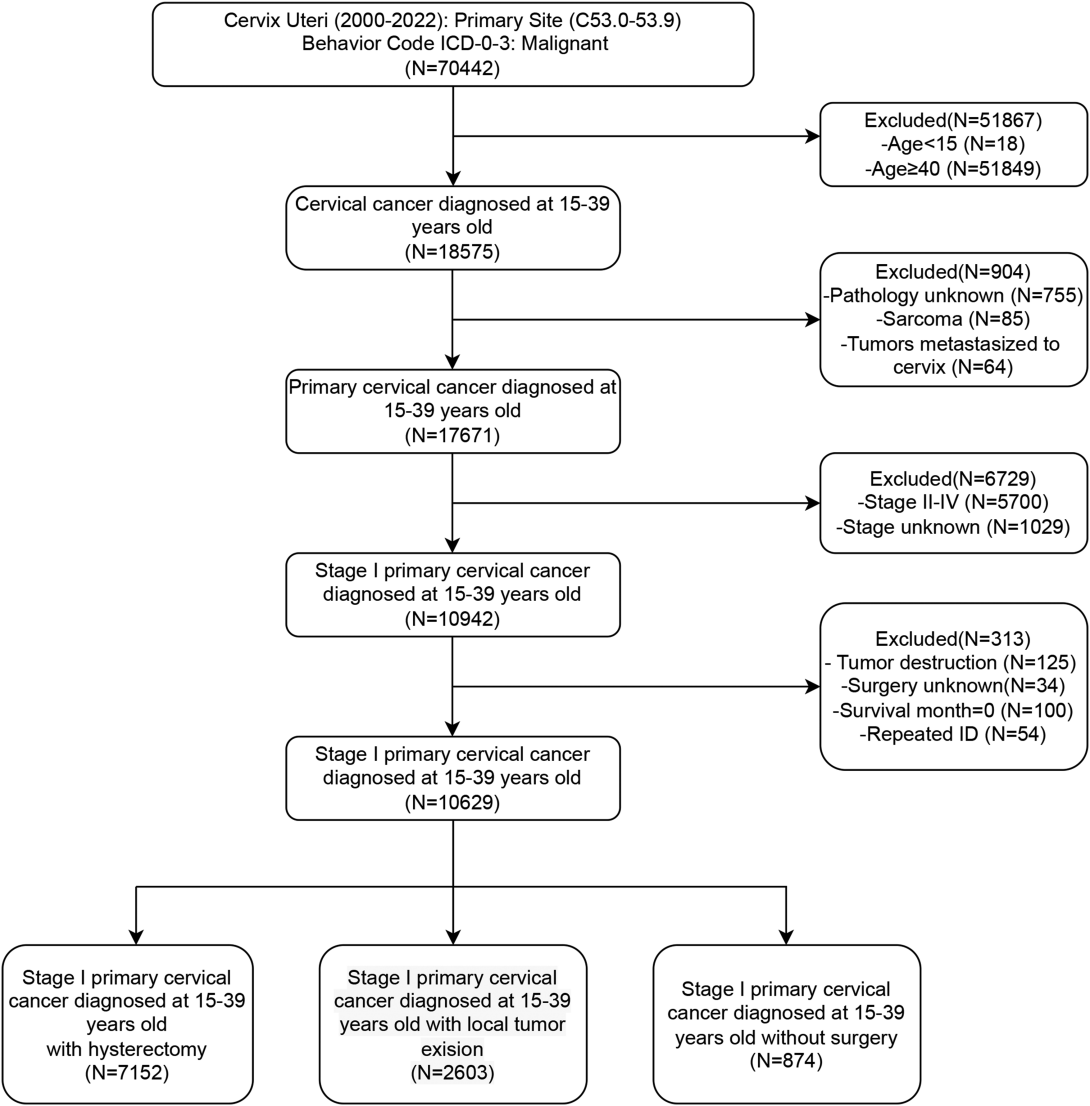
Screening flowchart.

Initially, patients with cervical lesions were identified by selecting the ICD-10 codes “C53.0–C53.9”. We subsequently collected basic patient information, including age, marital status, and ethnicity, as well as socioeconomic factors such as income and residence. Additionally, we gathered data on tumor characteristics, including pathological type, tumor differentiation, and FIGO staging, along with information regarding treatment and prognosis. Patients were categorized into four groups according to age: 15–24 years, 25–29 years, 30–34 years, and 35–39 years. Further stratification was performed based on marital status, dividing patients into married, single (including unmarried), or separated (including divorced, separated, or widowed) individuals. Ethnicity information primarily categorized patients into black, white, and other ethnic groups. Based on income status, patients were divided into three groups: high, medium, and low, with thresholds set at $35,000–$75,000 annually. Residential information stratified patients into urban and rural groups. The histological differentiation types were identified as squamous cell carcinoma (SCC), adenocarcinoma (AC), adenosquamous carcinoma (ASCC), and other subtypes. Patients were divided into Grade 1–2 (well and moderately differentiated) and Grade 3–4 (poorly differentiated and undifferentiated) groups according to their histological grade. Based on the description of surgical information, patients who underwent hysterectomy, local excision, or did not undergo surgery were included, excluding cases with only lesion destruction or inadequate surgical records. Local excision surgeries include “Local tumor excision, NOS”, “Local tumor excision with electrocautery”, “Local tumor excision with cryosurgery”, “Cone biopsy with gross excision of lesion”, “Dilatation and curettage; endocervical curettage (for *in situ* only)”, “Excisional biopsy, NOS”, “Cone biopsy”, “Cone biopsy with gross excision of lesion”, “Trachelectomy; removal of cervical stump; cervicectomy”. Hysterectomy surgeries include “Total hysterectomy”, “Modified radical or extended hysterectomy”, “Radical or extended hysterectomy”, “Hysterectomy, NOS”, and “Pelvic exenteration”. Survival indicators were collected, including survival time, survival status, and causes of death. Patients with missing survival data were excluded. Overall survival (OS) was defined as the time from diagnosis to death, whereas disease-specific survival (DSS) was defined as the time from diagnosis to death specifically attributable to cervical cancer. Duplicate patient IDs were eliminated to ensure data integrity. The detailed filtering process was shown in Figure 1.

### 2.3 Statistical analysis

Kaplan-Meier curves were generated to depict the prognosis of patients receiving different surgical interventions, and distinctions between these curves were evaluated through the log-rank test. Multivariate Cox regression analyses were applied to scrutinize the factors impacting prognosis, with the significance of these factors quantified by hazard ratios (HRs) and 95% confidence intervals (CIs). Statistical analyses and graphical representations were conducted using SPSS 26 and GraphPad Prism 9. A significance threshold of *P *< 0.05 was established to determine statistical significance.

## 3 Results

### 3.1 Patient clinical and pathological characteristics

We conducted a retrospective analysis of 10,629 stage I AYA cervical cancer patients between 2000 and 2020. Patients were categorized into three groups based on their surgical interventions: 67.3% of the patients (7,152 cases) underwent radical hysterectomy, 24.5% (2,603 cases) underwent fertility-preserving local excision, and 8.2% (874 cases) did not undergo any surgical intervention. The majority of patients (approximately 80%) were aged between 30 and 39 years. Notably, more than 50% of patients undergoing hysterectomy were in the 35–39 years group, while the highest proportion of those undergoing local excision were in the 30–34 years group. In terms of marital status, 54.2% of the patients who received a hysterectomy were married, suggesting that they may have already had children and experienced a decreased desire for fertility preservation. Conversely, a greater proportion of patients who underwent local excision were single or unmarried, indicating a greater desire for fertility preservation. Histologically, SCC was the most common subtype, accounting for 64.9%, followed by AC at 29.9%. ASCC and other histological types accounted for only 3.8% and 1.4%, respectively. In terms of FIGO stage, patients undergoing hysterectomy were evenly distributed in stages IA and IB, while the patients who underwent local excision were mostly in the earlier stage IA (72.1%), and the majority of those who did not undergo surgery were in the later stage IB (75.4%). Most of the patients who did not undergo surgery received radiotherapy and/or chemotherapy, whereas lower than 10% of the patients who underwent local excision received concurrent radiotherapy and/or chemotherapy (Table 1).

**Table 1:** Clinicopathologic profiles of stage I AYA cervical cancer with different surgery interventions.

Characteristics	Total *n* (%)	Hysterectomy *n* (%)	Local excision *n* (%)	None *n* (%)
Total	10,629 (100)	7,152 (100)	2,603 (100)	874 (100)
Age				
15–24	367 (3.5)	154 (2.2)	177 (6.4)	36 (4.1)
25–29	1,810 (17.0)	934 (13.1)	738 (28.4)	138 (15.8)
30–34	3,776 (35.5)	2,473 (34.6)	991 (38.1)	312 (35.7)
35–39	4,676 (44.0)	3,591 (50.2)	697 (26.8)	388 (44.4)
Marital state				
Marriage	5,210 (49.0)	3,878 (54.2)	1,009 (38.8)	323 (37.0)
Single	3,854 (36.3)	2,250 (36.1)	1,218 (46.8)	386 (44.2)
Separated	905 (8.5)	681 (9.5)	158 (6.1)	66 (7.6)
Unknown	660 (6.2)	343 (4.8)	218 (8.4)	99 (11.3)
Race				
Black	955 (9.0)	571 (8.0)	225 (8.6)	159 (18.2)
White	8,649 (81.4)	5,971 (83.5)	2,048 (78.7)	630 (72.1)
Others	864 (8.1)	543 (7.6)	261 (10.0)	60 (6.9)
Unknown	161 (1.5)	67 (0.9)	69 (2.7)	25 (2.9)
Income				
Low	84 (0.8)	64 (0.9)	8 (0.3)	12 (1.4)
Media	6,402 (60.2)	4,409 (61.6)	1,399 (53.7)	594 (68.0)
High	4,142 (39.0)	2,679 (37.5)	1,196 (45.9)	267 (30.6)
Rural/Urben				
Urben	9,470 (89.3)	6,317 (88.5)	2,410 (92.8)	743 (85.4)
Rural	1,132 (10.7)	819 (11.5)	186 (7.2)	127 (14.6)
Pathology				
SCC	6,893 (64.9)	4,399 (61.5)	1,821 (70.0)	673 (77.0)
AC	3,180 (29.9)	2,501 (32.5)	750 (26.9)	174 (18.2)
ASCC	406 (3.8)	318 (4.4)	61 (2.3)	27 (3.1)
Others	150 (1.4)	114 (1.6)	21 (0.8)	15 (1.7)
Grade				
1–2	5,033 (47.4)	3,710 (51.9)	1,082 (41.6)	241 (27.6)
3–4	2,080 (19.6)	1,600 (22.4)	282 (10.8)	198 (22.7)
Unknown	3,516 (33.1)	1,842 (25.8)	1,239 (47.6)	435 (49.8)
Stage				
IA	5,195 (48.9)	3,145 (44.0)	1,877 (72.1)	173 (19.8)
IB	5,238 (49.3)	3,913 (54.7)	666 (25.6)	659 (75.4)
I	196 (1.8)	94 (1.3)	60 (2.3)	42 (4.8)
Radiation				
No	8,592 (80.8)	5,993 (83.8)	2,354 (90.4)	245 (28.0)
Yes	2,037 (19.2)	1,159 (16.2)	249 (9.6)	629 (72.0)
Chemotherapy				
No	9,145 (86.0)	6,414 (89.7)	2,413 (92.7)	318 (36.4)
Yes	1,484 (14.0)	738 (10.3)	190 (7.3)	556 (63.6)

OS, overall survival; DSS, disease-specific survival.

### 3.2 Treatment choice and prognostic impact of surgery in stage I CC

**Figure 2:**
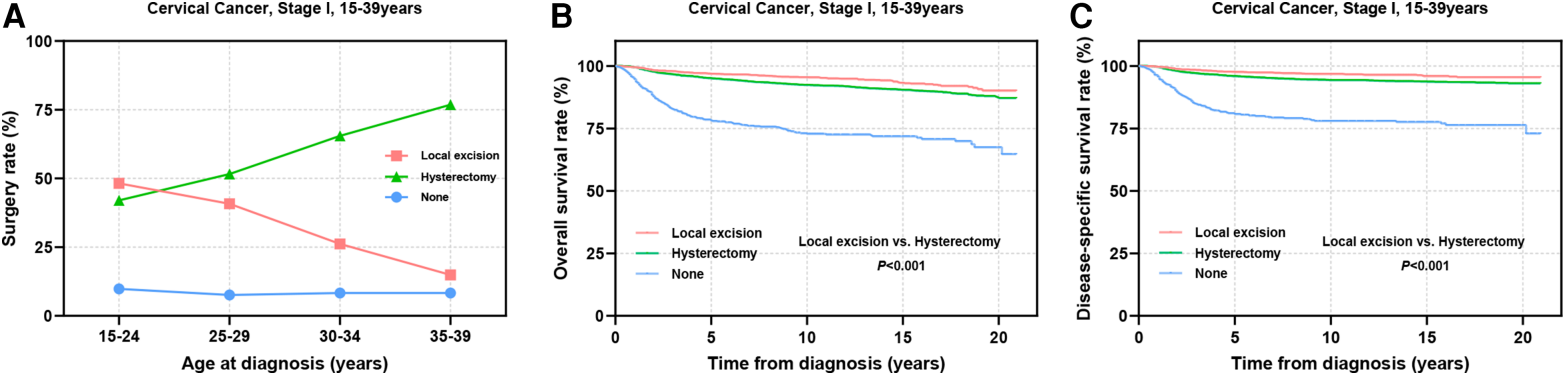
Prognosis of AYA stage I patients undergoing different types of surgical interventions. **(A)** The rate at which patients underwent local excision or hysterectomy; **(B**,**C)** OS and DSS of AYA stage I patients receiving local excision or hysterectomy.

Surgical intervention has emerged as the primary treatment modality for AYA patients with stage I CC (Figure 2A). Among this cohort, a greater proportion of individuals in the 15–24 years group underwent local excision (local excision: 48.2% vs. hysterectomy: 42.0%). As age increased, the adoption of local excision declined gradually, whereas the preference for hysterectomy rose steadily. Among patients aged 35–39 years, only 14.9% chose local excision, whereas 76.8% underwent hysterectomy (Figure 2A). Survival analysis revealed that among all stage I AYA patients, those who underwent local excision exhibited significantly superior OS and DSS outcomes in comparison to those who underwent hysterectomy (*P *< 0.001) (Figures 2B,C).

### 3.3 Factors affecting the prognosis of stage I CC patients

The prognostic analyses indicated favorable outcomes for local excision over hysterectomy as a treatment option for AYA with stage I CC. However, these findings pertain to the cohort as a whole, and may not necessarily apply to every individual seeking fertility preservation. Importantly, patients’ baseline characteristics, tumor attributes, and socioeconomic factors, play crucial roles as prognostic determinants. The different combinations of these factors for each patient may lead to distinct outcomes. Therefore, our study encompassed a range of variables, including age, marital status, race, income, residence, pathological subtype, histological differentiation, FIGO stage, and surgical approach, to conduct a comprehensive multivariate Cox regression analysis investigating the factors influencing OS and DSS in the patients. Our findings revealed that pathological subtype, differentiation, and stage were independent tumor factors influencing prognosis (Table 2). Upon adjusting for these variables, the choice between local excision and hysterectomy did not significantly impact OS (HR: 0.987; 95% CI: 0.8–1.218; *P *= 0.904) or DSS (HR: 1.033; 95% CI: 0.794–1.343; *P *= 0.810) in AYA patients with stage I CC.

**Table 2:** Multivariate Cox analysis of prognostic factors in stage I AYA cervical cancer patients.

Characteristics	OS		DSS	
	HR (95% CI)	*P*	HR (95% CI)	*P*
Age				
15–24	Reference		Reference	
25–29	1.25 (0.82–1.90)	0.309	1.16 (0.71–1.88)	0.556
30–34	1.39 (0.92–2.08)	0.114	1.23 (0.78–1.97)	0.376
35–39	1.42 (0.95–2.12)	0.092	1.06 (0.67–1.69)	0.798
Marital state				
Marriage	Reference		Reference	
Single	1.48 (1.26–1.73)	<0.001	1.30 (1.08–1.57)	0.006
Separated	1.54 (1.23–1.93)	<0.001	1.39 (1.05–1.84)	0.020
Unknown	1.51 (1.12–2.03)	0.007	1.29 (0.88–1.90)	0.192
Race				
Black	Reference		Reference	
White	0.77 (0.63–0.94)	0.009	0.67 (0.53–0.84)	0.001
Others	0.99 (0.73–1.34)	0.943	0.97 (0.69–1.36)	0.851
Unknown	0.18 (0.04–0.72)	0.015	0 (0–6.302 × 10^53^)	0.879
Income				
Low	Reference		Reference	
Media	0.65 (0.35–1.22)	0.179	0.69 (0.32–1.51)	0.349
High	0.57 (0.30–1.09)	0.089	0.66 (0.30–1.47)	0.308
Rural/Urben				
Urben	Reference		Reference	
Rural	1.07 (0.86–1.33)	0.563	1.00 (0.76–1.33)	0.975
Pathology				
SCC	Reference		Reference	
AC	0.71 (0.59–0.85)	<0.001	0.76 (0.61–1.00)	0.015
ASCC	1.69 (1.32–2.16)	<0.001	1.99 (1.51–2.61)	<0.001
Others	1.94 (1.27–2.97)	0.002	2.47 (1.59–3.82)	<0.001
Grade				
1–2	Reference		Reference	
3–4	1.37 (1.16–1.62)	<0.001	1.55 (1.28–1.88)	<0.001
Unknown	0.87 (0.72–1.06)	0.160	0.76 (0.60–0.98)	0.033
Stage				
IA	Reference		Reference	
IB	2.66 (2.21–3.19)	<0.001	4.50 (3.46–5.86)	<0.001
I	1.41 (0.86–2.34)	0.177	2.39 (1.26–4.52)	0.008
Surgery				
Hysterectomy	Reference		Reference	
Local excision	0.99 (0.80–1.22)	0.904	1.03 (0.79–1.34)	0.810
None	3.19 (2.68–3.80)	<0.001	3.50 (2.86–4.29)	<0.001

OS, overall survival; DSS, disease-specific survival; HR, hazard ratio; CI, confidence interval; SCC, squamous cell carcinoma; AC: adenocarcinoma; ASCC, adenosquamous cell carcinoma.

### 3.4 Surgical interventions for stage I CC patients with various pathological subtypes

**Figure 3:**
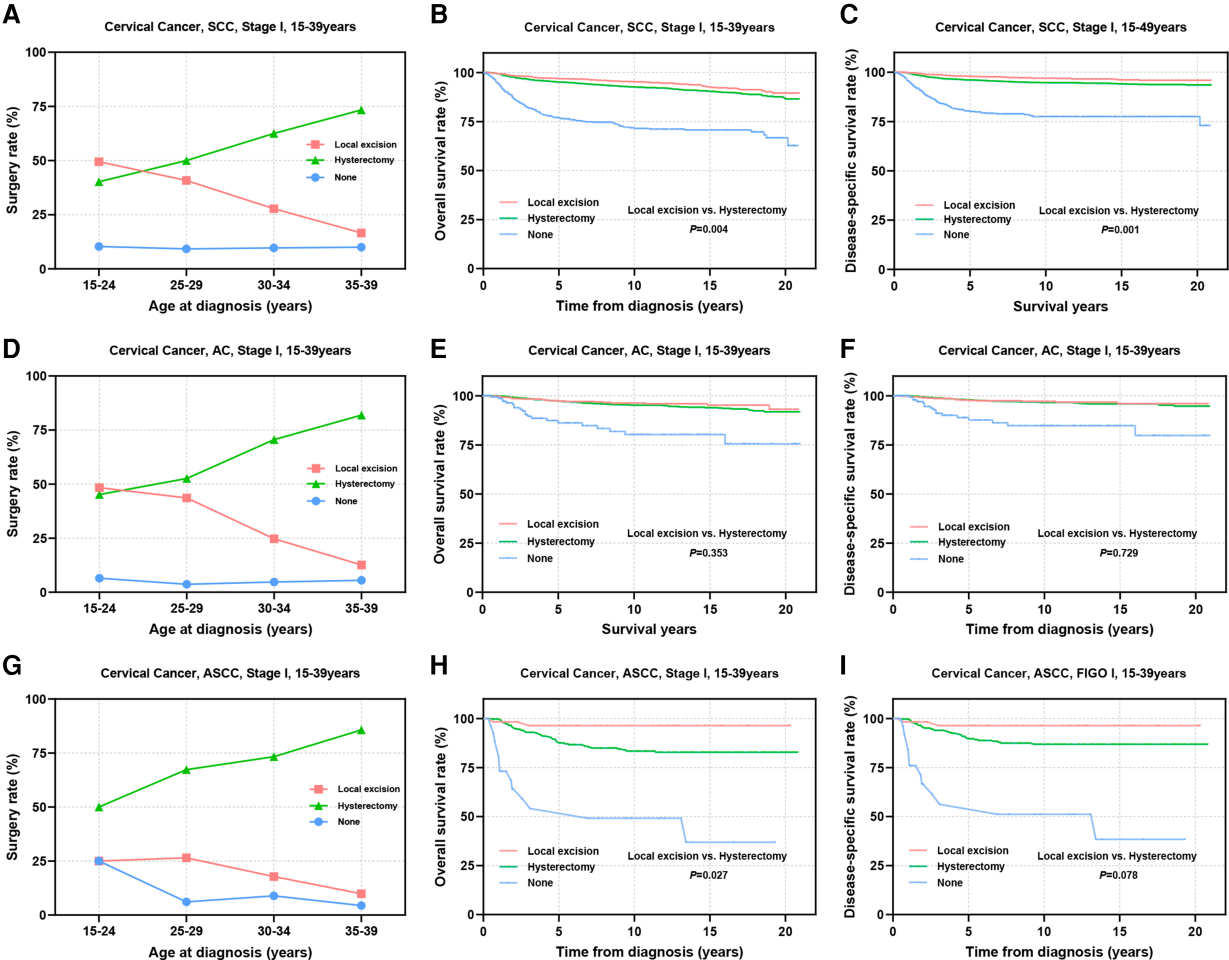
Prognosis of AYA stage I patients with different pathologic types receiving different types of surgical interventions. **(A**,**D**,**G)** Rates of patients with SCC, AC, ASCC receiving local excision or hysterectomy; **(B**,**C)** OS and DSS of stage I SCC patients undergoing local excision or hysterectomy; **(E**,**F)** OS and DSS of stage I AC patients undergoing local excision or hysterectomy; **(H**,**I)** OS and DSS of patients with stage I ASCC undergoing local excision or hysterectomy.

The multivariate Cox regression analyses highlighted the significance of pathological subtype, differentiation, and FIGO stage as independent factors influencing the prognostic outcomes of the patients. Subsequently, we delved into a stratified examination of these three tumor characteristics to explore potential differences in prognosis associated with distinct surgical interventions. Initially, we conducted separate analyses for SCC, AC, ASCC and other epithelial types of CC. Notably, the utilization of local excision tended to decrease with increasing age in patients with SCC and AC, dropping from approximately 50% in the 15–24 years group to 16.6% for SCC and 12.6% for AC in the 35–39 years group (Figures 3A,D). In contrast, the proportion of patients who underwent local excision remained consistently a low level for ASCC and other types across all age groups (ASCC: 9.9%–25.0%; others: 9.4%–24.3%) (Figure 3G, Supplementary Figure S1A). Prognostic analysis demonstrated that the therapeutic efficacy of local excision surpassed that of hysterectomy in patients with SCC (OS: 96.8% vs. 95.1%, *P *= 0.004; DSS: 97.9% vs. 96.0%, *P *= 0.001). A similar trend was observed in patients with ASCC (OS: 93.9% vs. 85.9%, *P *= 0.027; DSS: 93.9% vs. 87.8%, *P *= 0.078). Additionally, the treatment outcomes of local excision were comparable to those of hysterectomy in patients with AC (OS: 97.1% vs. 96.9%, *P *= 0.353; DSS: 97.5% vs. 97.6%, *P *= 0.729). Although the prognosis of ASCC is significantly lower than that of SCC and AC, the findings suggest that ASCC should not serve as a limiting factor for AYA with stage I CC to consider FSS (Figures 3B,C,E,F,H,I). In the context of other types of stage I CC, our analysis did not reveal a significant difference in efficacy between local excision and hysterectomy (Supplementary Figures S1B,C). However, it is important to note that the 5-year survival rate for other pathological types fell below 80%. Given the substantial surgical risks involved, the adoption of the FSS in patients with other histological types of CC warrants careful consideration and caution.

### 3.5 Surgical interventions for stage I CCs of different degrees of histological differentiation

**Figure 4:**
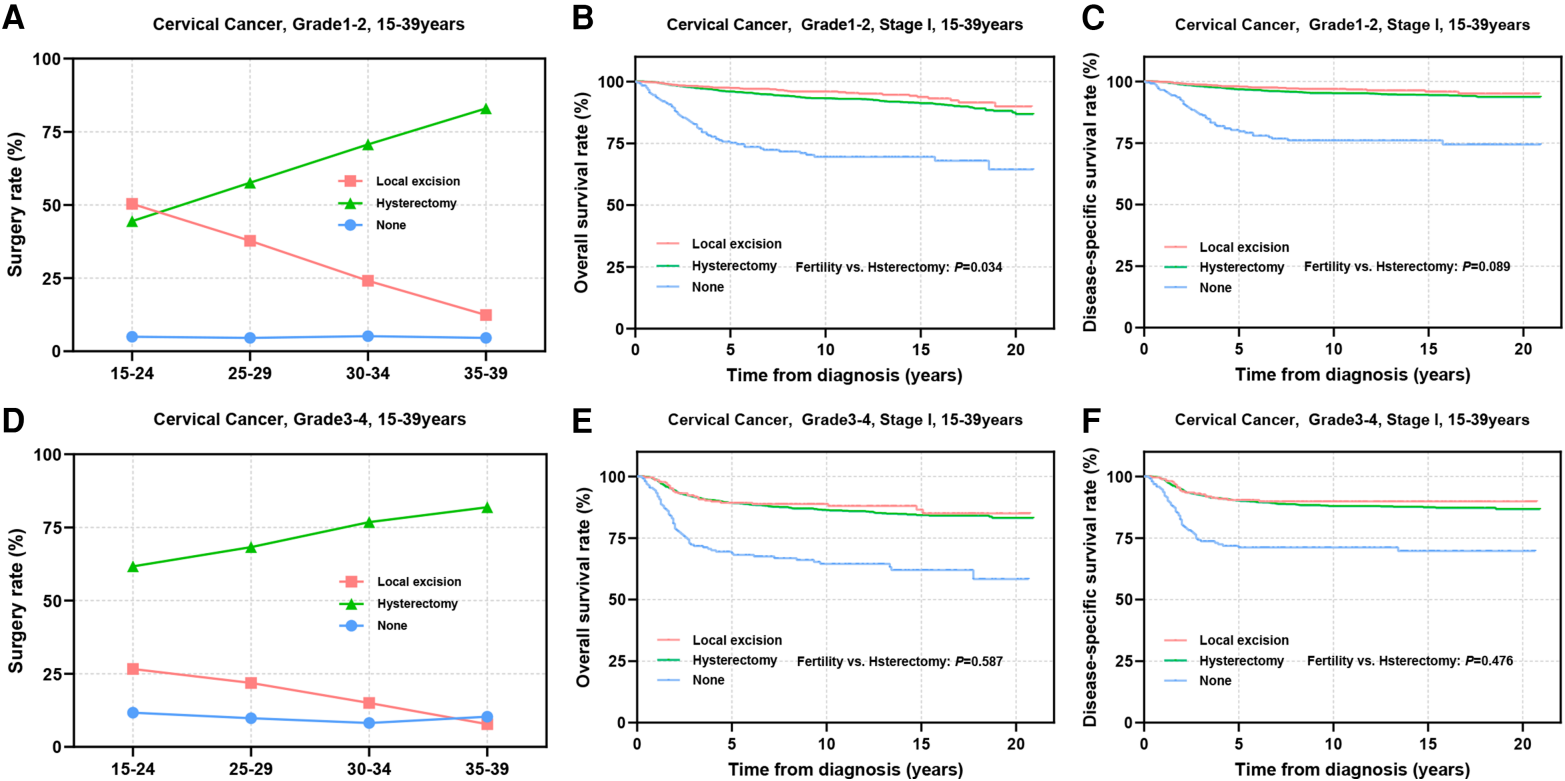
Prognosis in patients with different pathologic differentiations undergoing different types of surgical interventions. **(A**,**D)** The rate of Grade 1–2 **(A)** and Grade 3–4 **(D)** differentiated patients receiving local excision or hysterectomy; **(B**,**C**,**E**,**F)** OS and DSS of Grade1-2 **(B**,**C)** and Grade3-4 **(E**,**F)** differentiated patients undergoing local excision or hysterectomy.

Tumors with low differentiation often indicate an increased potential for metastasis and a less favorable prognosis. Subsequently, we conducted a stratified analysis on patients based on differentiation status. Among patients with well or moderately differentiated tumors, it was observed that younger individuals tended to undergo local excision, while the utilization of local excision in patients with grade 3–4 tumors was lower than that in patients with grade 1–2 tumors (Figures 4A,D). Prognostic analyses revealed that substituting hysterectomy with local excision surgery in patients with well or moderately differentiated tumors is a viable option (OS: 97.3% vs. 95.9%, *P *= 0.034; DSS: 97.8% vs. 96.8%, *P *= 0.089) (Figures 4B,C). Although, grade 3–4 differentiation status emerged as an independent factor influencing prognosis, the prognosis of patients who underwent different surgical procedures did not appear to be significantly influenced solely by differentiation status (OS: 89.2% vs. 89.3%, *P *= 0.587; DSS: 90.5% vs. 90.2%, *P *= 0.476) (Figures 4E,F).

### 3.6 Surgical interventions for stage I CC patients at different tumor stages

**Figure 5:**
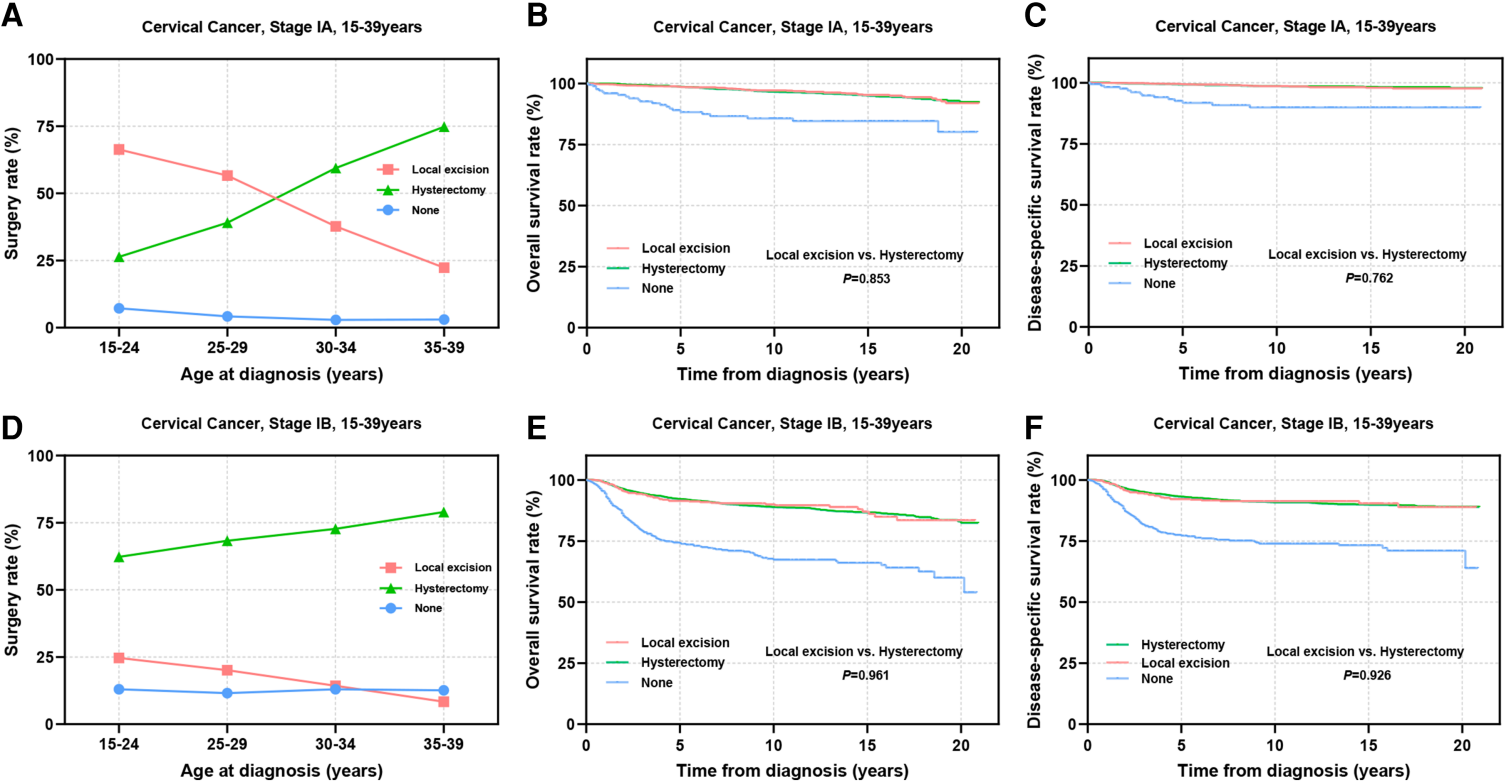
Prognosis in AYA stage IA and IB patients undergoing different types of surgical interventions. **(A**,**D)** The rate of stage IA **(A)** and stage IB **(D)** patients receiving local excision or hysterectomy; **(B**,**C**,**E**,**F)** OS and DSS of stage IA **(B**,**C)** and stage IB **(E**,**F)** patients undergoing local excision or hysterectomy.

The stage of cervical cancer plays a crucial role in determining prognosis and guiding surgical decision-making. Subsequently, we performed a stratified analysis on patients at stage IA and IB. Among patients at stage IA, the percentage of patients receiving local excision was notably high at 66.3% in the 15–24 years group, this percentage gradually declined to 22.3% with age advancing (Figure 5A). Conversely, for patients at stage IB, the adoption of local excision remained relatively low across all age groups (8.4%–24.7%) (Figure 5D). In the stage IA group, there were no significant disparities in OS (98.5% vs. 98.6%, *P *= 0.853) and DSS (99.4% vs. 99.2%, *P *= 0.762) between patients undergoing local excision and those receiving hysterectomy (Figures 5B,C). Similarly, among patients at stage IB, no statistically significance was observed in OS (91.4% vs. 92.2%, *P *= 0.961) and DSS (92.1% vs. 93.2%, *P *= 0.926) between the two surgical choices (Figures 5E,F).

**Figure 6:**
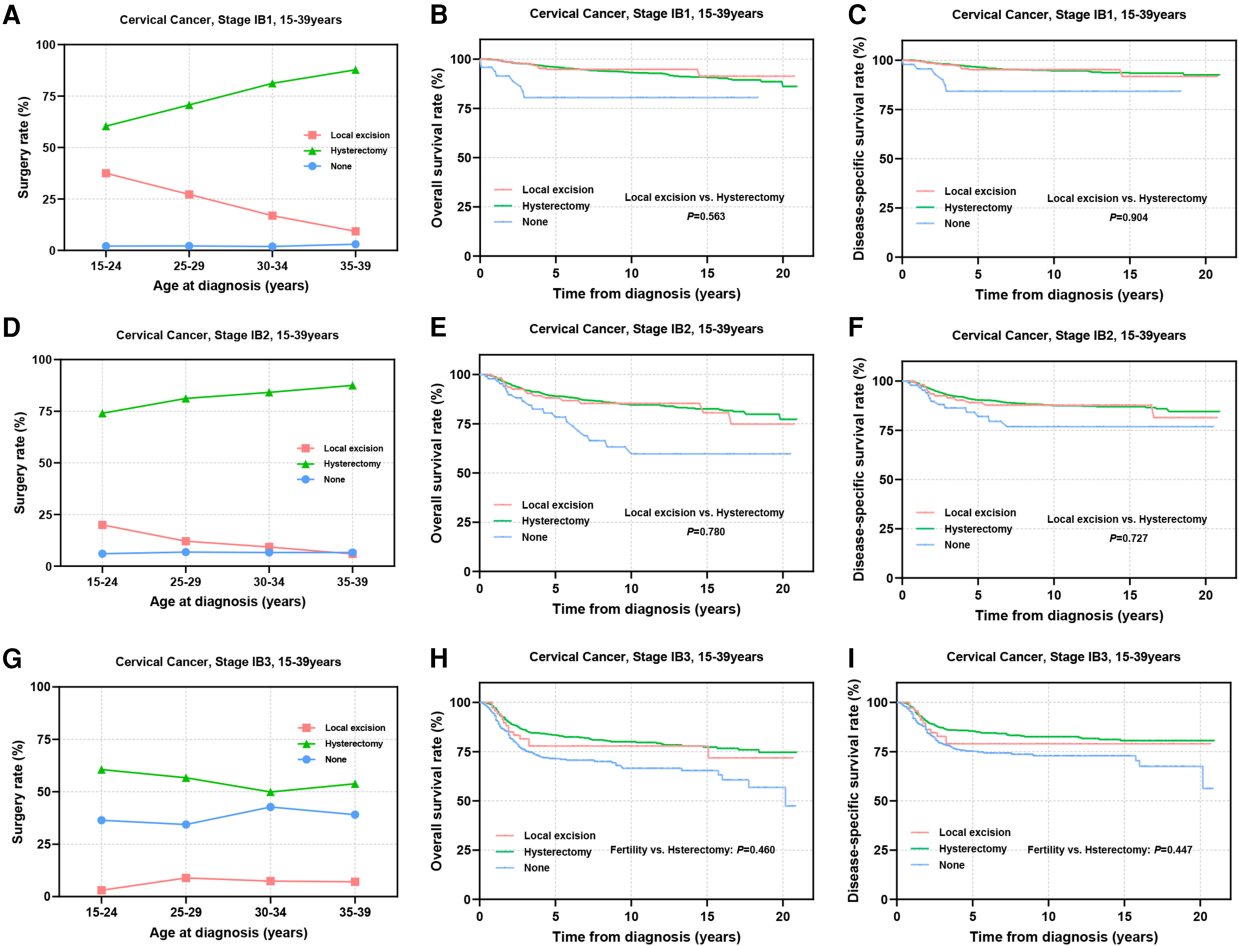
Prognosis of stage IB1-IB3 AYA patients undergoing different types of surgical interventions. **(A**,**D**,**G)** Rates of patients at stage IB1, IB2, IB3 receiving local excision or hysterectomy; **(B**,**C)** OS and DSS of stage IB1 patients undergoing local excision or hysterectomy; **(E**,**F)** OS and DSS of stage IB2 patients undergoing local excision or hysterectomy; **(H**,**I)** OS and DSS of patients with stage IB3 undergoing local excision or hysterectomy.

The international application of FSS for CC patients remains controversial, primarily concerning the tumor size threshold. ESGO 2023 suggests that patients with SCC and HPV-related AC with a maximal tumor diameter ≤2 cm may be considered for FSS, while the National Comprehensive Cancer Network (NCCN) guidelines propose the possibility of fertility preservation for select stage IB2 (2 cm < diameter ≤ 4 cm) patients (12, 13). Thus, we further subdivided stage IB into IB1–IB3 for analysis. The results showed that the proportion of stage IB1 patients receiving local excision ranged from 9.3% to 37.5% (Figure 6A). For patients in IB2 stage, thia percentage decreased to 5.9%–20% (Figure 6D). Only 3.0%–8.9% of patients in IB3 stage underwent FSS (Figure 6G). Prognostic evaluation indicated comparable 5-year survival rates between the local excision and hysterectomy for stage IB1 patients (5-year OS: 94.7% vs. 95.9%, *P *= 0.563; 5-year DSS: 95.2% vs. 96.5%, *P *= 0.904) (Figures 6B,C) and stage IB2 patients (5-year OS: 88.0% vs. 89.0%, *P *= 0.780; 5-year DSS: 89.1% vs. 90.3%, *P *= 0.727), suggesting that FSS may be considered for stage I patients with tumor diameters of 2–4 cm (Figures 6E,F). In IB3 patients, although there was no statistically significant difference in long-term survival rates and DSS between the two surgical interventions (Figures 6H,I), patients undergoing local excision showed significantly reduced 5-year OS and DSS (5-year OS: 77.8% vs. 83.4%; 5-year DSS: 79.0% vs. 85.5%). Therefore, careful consideration is imperative for stage IB3 AYA patients opting for local excision.

### 3.7 Causes of death in AYA stage I CC patients

**Figure 7:**
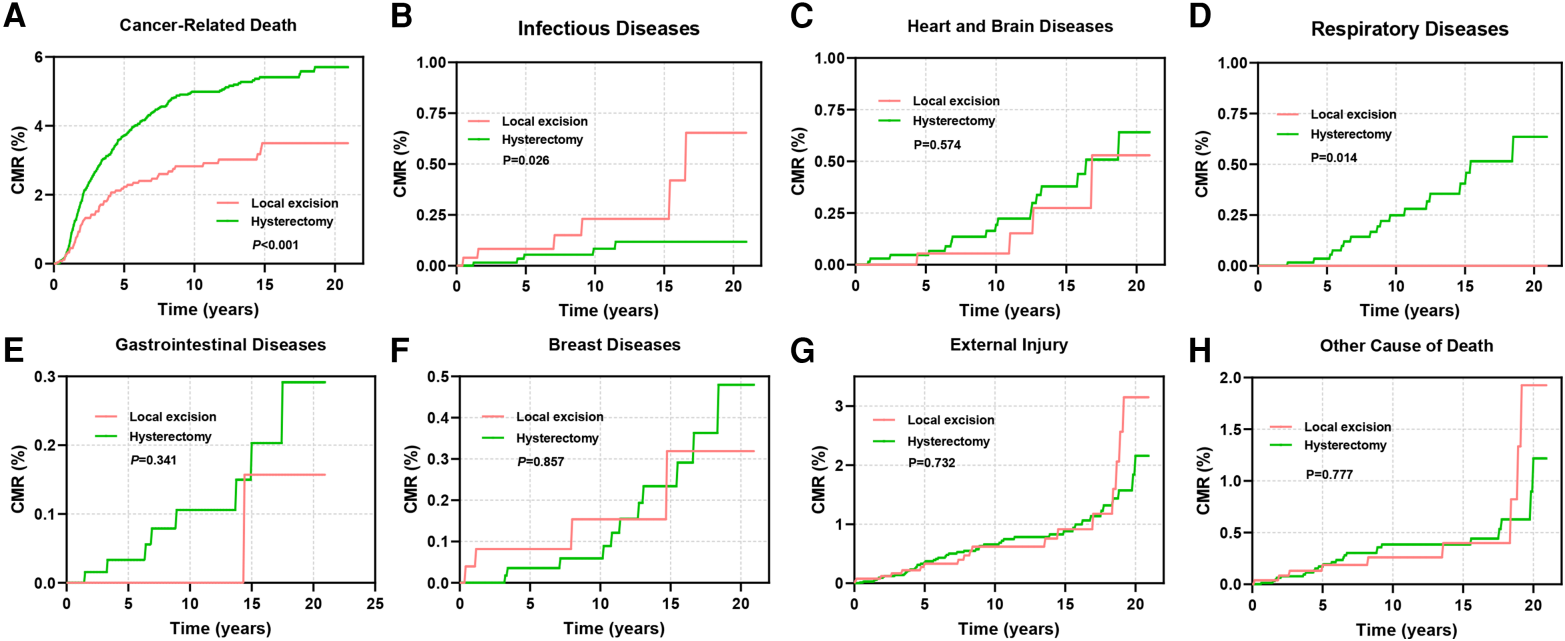
CMR among AYA stage I patients undergoing different types of surgical interventions. **(A)** CMR due to cancer-related diseases; **(B)** CMR due to infectious diseases; **(C)** CMR due to diseases of the heart and brain; **(D)** CMR due to respiratory diseases; **(E)** CMR due to gastrointestinal diseases; **(F)** CMR due to breast diseases; **(G)** CMR due to external injury; **(H)** CMR due to other causes of death.

This study also investigated the cumulative mortality rates (CMRs) attributed to diverse causes of death in cervical cancer patients (Figures 7A–H). Patients undergoing local excision exhibited notably lower rates of cancer-related mortality in comparison to those undergoing hysterectomy (*P *< 0.001, Figure 7A). However, the mortality rate stemming from infectious diseases was significantly elevated in the former group (*P *= 0.026, Figure 7B). Patients receiving hysterectomy displayed a heightened CMR linked to gastrointestinal and cardiovascular ailments during the initial phases of follow-up, although these differences did not reach statistical significance (Figures 7C–H).

## 4 Discussion

With the increasing trend of conceiving age, the demand for fertility preservation in early-stage CC patients has gained prominence. The recommendation of FSS remains to be more individualized and precise. This study delved into the interventions and outcomes of 10,629 AYA with stage I CC. In this cohort, approximately one-third of the patients opted for FSS, with this percentage increasing to nearly 50% among patients aged 15–24 years. Our study demonstrated that the outcomes of local excision surgery were comparable to those of hysterectomy in the whole AYA stage I CC patients, aligning with findings of Ying Chen's study (4). However, variations in pathological type, tumor differentiation, and FIGO stage influenced the surgery choices of patients.

SCC stands as the predominant histological type of CC, followed by AC, ASCC, and others (14, 15). With the widespread adoption of cervical screening and HPV vaccination, the incidence of SCC has gradually declined, while the prevalence of AC has been increasing (14). Studies have suggested a poorer prognosis associated with AC and ASCC compared to SCC (16–18), although some studies have reported no discernible difference in prognosis upon considering tumor staging (19, 20). Our study specifically investigated stage I cervical cancer patients aged 15–39 years and found that the prognosis of patients with AC is significantly better than that of patients with SCC, whereas ASCC is associated with a worse prognosis than SCC. This disparity in prognosis also influences the choice of FSS. Notably, young patients with cervical ASCC are notably less likely to choose local excision compared to those with SCC or AC. Even within the 15–24 years group, only 25% of patients choose FSS, whereas the percentage among patients with SCC and AC is nearly 50%. Further prognosis analyses suggested that patients with SCC may derive greater benefit from local excision. For patients with AC, local excision is a viable option since hysterectomy has not shown superior survival outcomes. In the case of ASCC patients, although their prognosis may be less favorable than that of SCC patients, there is no evidence indicating that FSS negatively impacts the prognosis of ASCC patients. While additional adjuvant therapy may be necessary based on the specific pathology, ASCC should not be considered a limiting factor in the decision-making process concerning fertility preservation for AYA stage I patients.

Histological differentiation appeared to affect decision-making regarding fertility preservation. Fower than 15% of patients with poorly differentiated or undifferentiated tumors chose FSS. Safety evaluations suggested that patients with well or moderately differentiated (grade 1–2) tumors tend to benefit more from local excision. In patients with poorly differentiated and undifferentiated tumors, local excision led to comparable outcomes with hysterectomy. We believe that tumor differentiation should not be a decisive factor for AYA stage I patients considering FSS, especially among patients with SCC, AC, or ASCC histopathological types. However, lower differentiation is often accompanied by an advanced stage at the time of initial diagnosis. This correlation may have influenced the adoption of FSS for patients with poorly differentiated CC.

Tumor stage is a key factor affecting the prognosis of CC patients and is also an important factor for considering the FSS in young CC patients. In our study, more than 50% of AYA patients at stage IA underwent local excision surgery. Concerns regarding the impact of stage progression on prognosis affect the choice of fertility preservation. The proportion of stage IB2-IB3 AYA patients receiving local excision experienced a sharp decline. Previous studies and guidelines such as the NCCN 2024 and the ESGO have consistently confirmed the safety and feasibility of FSS in stage IA-IB1 patients (12, 13, 15, 21, 22). Similarly, our study in a larger cohort validated the viability of local excision as an alternative to hysterectomy for preserving fertility in stage I AYA patients. However, recommendations for fertility preservation in stage IB2-IB3 patients remain controversial. The safety and efficacy of neoadjuvant chemotherapy combined with local excision as a treatment option for preserving fertility have been gradually explored in early-stage CC patients. A meta-analysis showed that neoadjuvant chemotherapy combined with local excision was a viable option for fertility preservation in patients with a tumor diameter of 2–4 cm, with an efficacy rate of 92% and a postoperative recurrence rate of 6.1% (23). The ongoing prospective CONTESSA study (NCT 04483557) plans to include patients with tumor sizes of 2–4 cm who wish to preserve fertility, exploring the option of FSS for patients who have achieved complete or partial response after neoadjuvant chemotherapy (residual lesion <2 cm), and the results will be ready by 2025 (24). In our research, we demonstrated that local excision surgery had comparable prognostic outcomes to hysterectomy in a cohort of more than 1,400 patients at stage IB2. The high recurrence rate reported in some other studies of stage IB2 patients may be due to the tumor itself rather than the choice of surgery type. Recent studies have confirmed notable pregnancy and live birth rates following FSS in stage IB2 patients (25, 26). Overall, FSS was feasible in stage IA-IB2 AYA patients. For patients at stage IB3, consistent with most studies and guideline recommendations, our study revealed that the 5-year survival rate of patients who underwent local excision surgery was significantly lower than that of patients who underwent hysterectomy in these patients, even though adjuvant chemotherapy was very common in this patient population. Therefore, careful consideration is warranted regarding FSS for stage IB3 patients.

Comprehensive preoperative and intraoperative evaluations are needed for implementing FSS. It is crucial to utilize pelvic MRI, PET-CT, and other examinations to evaluate lymph node involvement and deep stromal tissue infiltration. Lymphovascular space invasion (LVSI)is a risk factor for lymph node metastasis and indicates a poor prognosis in early-stage cervical cancer (27). For stage IA patients without LVSI, lymph node dissection is optional, while for patients with LVSI, pelvic lymph node dissection or sentinel lymph node (SLN) assessment is imperative (12, 27). In early-stage cervical cancer, SLN evaluation has been shown comparable prognostic value to traditional pelvic lymph node dissection (28). Both the ESGO guidelines and the NCCN guidelines recognize the effect of SLN assessment in lymph node evaluation for FSS (12). These evaluations play a pivotal role in identifying individuals at high risk of recurrence and ensuring their exclusion from FSS (2, 29). The high incidence of complications associated with radical surgeries has led to a shift toward less radical surgery for early-stage, low-risk CC, offering fertility-sparing options for patients (30). In our study, we observed that patients with early-stage, well- or moderately-differentiated SCC may benefit from local excision surgery, even if fertility preservation is not the primary goal. For the selection of surgical paths, the minimally invasive surgical approach may be more preferable to the laparotomic approach, for it can reduce the occurrence of complications and improve the postoperative pregnancy rate without increasing the recurrence rate (18, 31). Following surgery, vigilant monitoring and consideration of adjuvant radiotherapy or chemotherapy based on tumor characteristics are important for improving patient prognosis (32–35).

While our study provides insights, it has limitations. Retrospective analysis, despite its large case count, can only offer evidence for local excision as a viable alternative to hysterectomy for FSS in AYA stage I CC patients. Prospective studies are necessary for definitive guidance. Second, the absence of detailed information on specific surgical approaches, extent of surgery and lymphovascular invasion information in the database, as well as surgical heterogeneity due to longer study timeline, impedes a more comprehensive assessment of the prognostic impact of various surgical choices. Third, while the ultimate objective of FSS is to maintain fertility potential, the database also lacks long-term follow-up data, posing challenges in evaluating the final fertility outcomes across different age groups, tumor characteristics, and surgical interventions.

## 5 Conclusion

The pathological type, low degree of differentiation, and relatively advanced tumor stage limit the choice of fertility preservation for AYA patients with stage I CC. In AYA patients with stage IA or IB2, the efficacy of local excision surgery and hysterectomy was comparable. Tumor differentiation should not be a restrictive factor for fertility preservation in stage I AYA patients.

## Data Availability

The raw data supporting the conclusions of this article will be made available by the authors, without undue reservation.
