# Supplementary material for: Adoption strategies of fertility-sparing surgery for early-stage cervical cancer patients based on clinicopathological characteristics: a large retrospective cohort study
Source: Front Surg. 2024 Aug 22;11:1456376. doi: 10.3389/fsurg.2024.1456376 (PMC11375612; doi:10.3389/fsurg.2024.1456376)
Supplement: Supplementary file 1 [file Datasheet1.pdf]

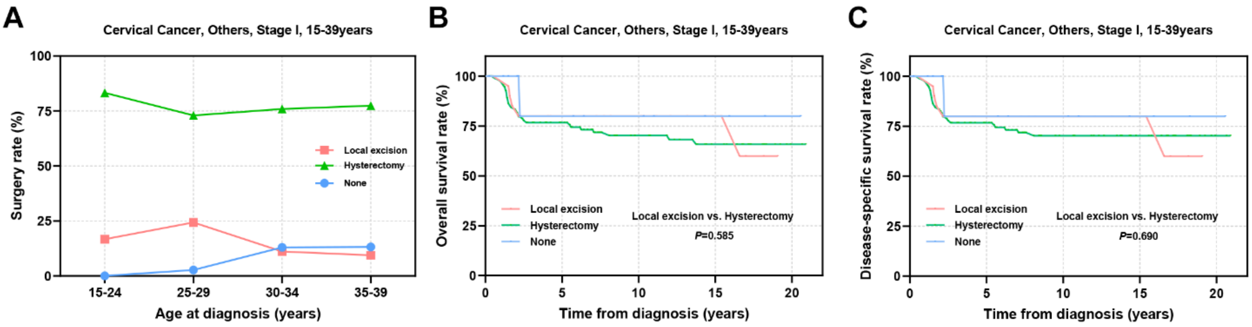


Fig.S1 (A) Rates of patients with other pathological types receiving local excision or hysterectomy; (B, C) OS and DSS of other pathological types in stage I patients receiving local excision or hysterectomy.
